# The Analysis of Mathematics Academic Burden for Primary School Students Based on PISA Data Analysis

**DOI:** 10.3389/fpsyg.2021.600348

**Published:** 2021-02-10

**Authors:** Li Wang

**Affiliations:** Curriculum and Teaching Materials Research Institute, People’s Education Press, Beijing, China

**Keywords:** primary school students, mathematics burden, mathematics anxiety, PISA2018, mathematical literacy

## Abstract

To explore the impact of academic burden on the physical and mental health of primary school students, combined with the results of the Programme for International Student Assessment (PISA) report in 2018, the relationship among the development of mathematical literacy, mathematics academic burden, and the physical and mental health of primary school students is studied. First, the relationship between mathematical literacy and mathematics anxiety is analyzed, and related influencing factors and measurement methods of mathematics anxiety are introduced. A questionnaire is then designed for primary school students’ mathematical stress, and the reliability and validity of the designed questionnaire are tested. Finally, a questionnaire survey is conducted on students, parents, and teachers in the third, fourth, and fifth grades of three standardized public primary schools. The results of the questionnaire survey show that students, teachers, and parents have a general understanding of the mathematics academic burden of primary school students at this stage. A total of 70% of teachers believe that primary school students have a heavy mathematics burden; 50% of parents think that primary school students are under heavy academic stress; 70% of primary school students believe that the heavy mathematics burden leads to reduced sleep time and extracurricular activities, which has a serious impact on the physical and mental health of primary school students. This research provides a reference for improving the current balance between education and students’ physical and mental health in China.

## Introduction

The Programme for International Student Assessment (PISA) report results in 2018 show that the average on-campus learning time of students in four provinces in China is 31.8 h per week, ranking 4th among countries (regions) participating in the test. For individual learning time, the average reading, mathematics, and science learning time of students in the four provinces in China is 4.6 h per week, 5.0 h per week, and 5.5 h per week, respectively, ranking the 7th, 8th, and 3rd among countries (regions) participating in the test. For the total learning time, students in the four provinces of China spend more time on reading, mathematics, and science. The class hours of the three subjects account for 47.6% of the total class hours, ranking 17th among countries (regions) participating in the test (Pino et al., 2016). It also reveals the problems that exist in current Chinese mathematics teaching (Estévez et al., 2018). Health refers to good physical, mental, moral, and social adaptability. With the deepening attention to students’ mental health, it is found that students’ mental health shows a downward trend, showing bipolarity and instability in psychological problems (Tsocheva et al., 2018).

Quis (2018) explored the impact of reducing high school curriculum time and increasing teaching intensity on mental health and mental stress of students in schools in the German state Baden-Wurttemberg. The questionnaire information of 2,306 students was also analyzed. The results show that the measures have a strong negative impact on students’ mental health problems, increasing their learning stress (Quis, 2018). Yang and Fan (2019) analyzed PISA data and the analysis report released by the Organization for Economic Co-operation and Development since 2009. It was concluded that Chinese education faced the problem of a heavy burden of school students and the increase of extracurricular expenses. They analyzed the problems of digital governance caused by PISA and the problems in the reform of China’s education system (Yang and Fan, 2019). Subramani and Kadhiravan (2017) collected data from 200 high school students in public and private schools in Salem City, Tamil Nadu through stratified random sampling and explored the relationship between academic stress and mental health of high school students. The results show that academic stress has a significant relationship with the mental health of high school students.

To explore the correlation between mathematics academic burden and the physical and mental health of primary school students, the results of the PISA report in 2018 was analyzed to obtain the current status of the academic burden of Chinese students. The relationship between students’ mathematical literacy and mathematics anxiety was also studied to analyze the factors that cause students’ mathematics anxiety. A related questionnaire was designed to investigate the students, parents, and teachers’ understanding of students’ mathematics academic burden. The impact of excessive mathematics academic burden on the development of primary school students’ physical and mental health was explored to provide effective solutions to students’ physical and mental stress.

## Materials and Methods

### Analysis of the Correlation Between Mathematical Literacy and Mathematical Anxiety

The primary school period is an important stage for cultivating students’ independence, innovative thinking, and sound personality. However, the cultivating process is suppressed under the influence of excessive academic burden, which has a serious negative impact on the cultivation of students’ view of life and values. In the mathematics curriculum, it emphasizes the cultivation of students’ mathematical literacy, which focuses on the ability to use mathematics to solve problems in real situations. The PISA test applies many scenarios to allow students to find resonance in the test questions. Therefore, what the PISA assesses is not the student’s proficiency and mastery of the subject, but the student’s readiness for various areas of life (Herborn et al., 2020).

Moreover, due to the imperfect physical and mental development of students, their emotional regulation ability is weak, which affects their physical and mental health. Moreover, students face huge academic stress and academic burden, which leads to different subjective attitudes among students; some students have poor psychological adjustment abilities and show learning anxiety in the face of stress, which affects their healthy development. Therefore, studying the students’ attitude toward academic burden can improve students’ attitude toward learning from the perspective of psychology, so that students can enjoy learning (Wu et al., 2019). Based on this, the relationship between mathematical literacy and mathematics anxiety is explored based on the PISA results to further study the correlation between academic burden and students’ physical and mental health.

**FIGURE 1:**
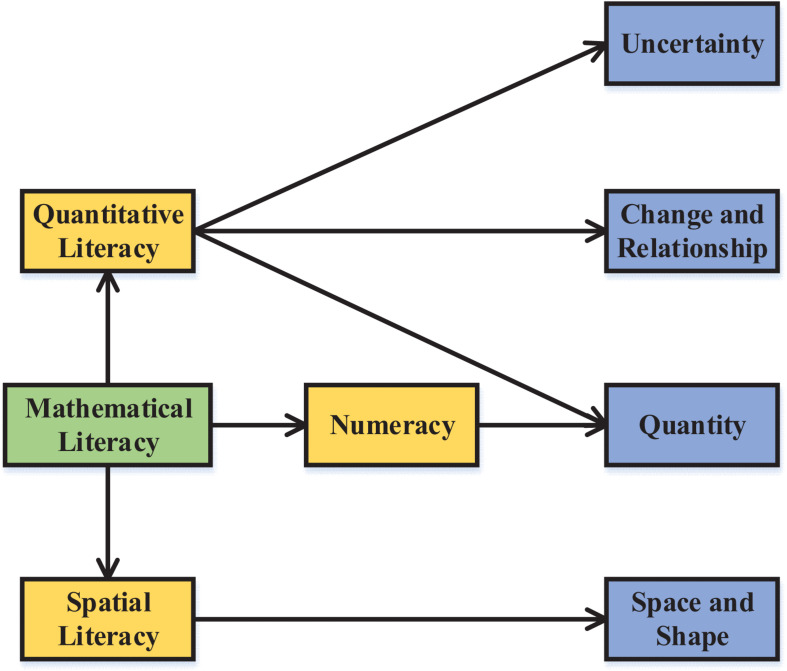
The relationship among Numeracy, QL, and ML.

Mathematical literacy is a basic quality that everyone should have in the development of modern society. It refers to an ability cultivated by an individual in the process of learning mathematics, including conceptual understanding, strategic ability, process fluency, reasonable reasoning awareness, and positive inclinations. China has ranked among the best in several PISA tests. The result has aroused a strong response from education circles worldwide, and people gradually began to pay attention to Chinese education circles. In the PISA test, the individual, society, and mathematics are combined and interacted to construct a unique evaluation framework, which is carried out from the three dimensions of mathematical content, mathematical problems, and mathematical processes. PISA stimulates students’ mathematical literacy through test questions. The real problems are converted into the actual application process of students’ mathematical thinking to judge their level of mathematical literacy (Marconnot et al., 2019). On the contrary, through the analysis of PISA results, it can be understood how to improve students’ mathematical literacy and provide new methods for the correct understanding of mathematical literacy. Numeracy, Quantitative Literacy (QL), and Mathematical Literacy (ML) are often used worldwide to describe mathematical literacy. Sometimes, the three are even used interchangeably. The relationship between the three is shown in Figure 1 (Montes, 2019; O’Brien et al., 2020).

The definition of mathematics anxiety is not fixed, but it can have an impact on students’ academic performance, which is recognized by research circles worldwide. The specific manifestation is that students will show various anxiety states when they encounter mathematical problems in the learning process, which will cause their academic performance to gradually deteriorate. Later investigations have shown that when dealing with life problems and learning situations, individuals with mathematics anxiety cannot solve their anxiety through familiarity or mathematical operations. The investigation of learning anxiety is taking place relatively late in China. It is generally believed that learning anxiety is a special subject anxiety disorder, which is a negative cognitive emotion (Hopp, 2019; Simamora and Saragih, 2019; Guo et al., 2020).

For the measurement methods of mathematics anxiety, a few scholars use questionnaires designed by themselves, and most use existing mathematics anxiety scales. The Mathematics Anxiety Rating Scale (MARS) of Richardson and Suinn is mostly used (Huang et al., 2020). This scale is used to evaluate the mathematics anxiety level of college students. There are 98 questions, and the internal consistency coefficient is 0.97. Most of the other mathematics anxiety scales are obtained by modifying MARS (Cairns et al., 2019; Ferrari et al., 2020; Li et al., 2020; Wang et al., 2020).

The investigation of mathematics anxiety worldwide focuses mainly on the following aspects: (1) the relationship between academic performance and mathematics anxiety; (2) the relationship between student gender and mathematics anxiety; (3) the relationship between the grade and mathematics anxiety; and (4) the relationship between self-efficacy and mathematics anxiety. The results obtained, however, are not uniform or even contradictory (Zhou et al., 2020). The results of PISA show that when comparing students worldwide, the higher the mathematics anxiety, the lower the score of mathematical literacy. In most cases, students’ mathematics anxiety is caused by excessive mathematics learning burden.

The current overburden of primary and secondary school students has become an important issue affecting the overall development of education in China. It is related to whether China can cultivate talents with an innovative spirit and practical ability (Sellers et al., 2019). The essence of education is to follow human nature. However, under the influence of society, parents, and schools, students can only passively accept some heavy learning burdens, including learning requirements that exceed the curriculum standards, extended learning time, and excessive homework (Huston et al., 2019). It has resulted in students’ schoolbags becoming increasingly heavier in recent years, and there are more cram schools after class and less time to sleep. In addition, the instillation of parents’ educational philosophy has caused the increasing stress on students, which has severely suppressed and destroyed the cultivation of students’ independence, sound personality, and innovative thinking. It has a negative impact on the establishment of their outlook on life, values, and morals (Pedersen et al., 2019; Cozzolino et al., 2020).

### Design of Questionnaire Survey on Academic Stress of Primary School Students

The life style of primary school students is simple, the scope of activities is mainly school and home, and the learning stress mainly comes from school and parents (Moyano et al., 2020). To understand the real situation of primary school students’ mathematical academic burden, it must be looked at from three aspects – students, parents, and teachers – to find the root of the current situation of primary school students’ burden, and to put forward improvement suggestions and feasible measures and to discuss the impact of heavy mathematics academic stress on primary school students’ physical and mental health (Romano et al., 2020).

The questionnaire survey is conducted on three standardized public primary schools in City A. To understand the situation from the perspective of the students, especially the situation of those with immature ideological development in the lower grades, students in the third, fourth, and fifth grade, teachers, and their parents were surveyed. The scientific method was used to conduct the survey to ensure the reliability of the data obtained. To better understand the current situation of primary school students’ academic burden, through the study and induction of related theories, the questionnaire on primary school students’ academic burden was designed from four aspects; learning time, schoolwork quality, learning stress, and schoolwork difficulty.

(1) Learning time. The class time, homework time, the time for making up missed lessons, sleep time, as well as time for independent learning and free activities outside of class are investigated. A survey of teachers’ classroom time was also conducted.
(2) Schoolwork quality. The effectiveness of the school’s curriculum, homework, and examinations, as well as the students’ ideas, are investigated. The effectiveness of school teaching affects students’ acceptance of what they learn. If the teacher repeats the mechanical training, it will increase the student’s mathematics learning burden. Therefore, the analysis is carried out from the aspects of learning styles, teaching styles, test content, and teachers’ after-school guidance. Curriculum standards are a reference index for measuring the quality of teaching and evaluating the quality of teaching courses (Carpenter et al., 2019; Tur-Porcar et al., 2019).
(3) Learning stress. The various emotions expressed by students in the process of mathematics learning are investigated. The concrete manifestation of their learning burden is their subjective feelings in the learning process, which are obtained through the evaluation of students, parents, and teachers. The survey was conducted for the students’ learning attitude, homework attitude, the attitude towards making up missed lessons, test attitude, and self-expression. Further, the attitude of parents and teachers toward students’ extracurricular tutoring was also investigated (Hoagwood et al., 2020).
(4) Schoolwork difficulty. It refers to the difficulty of teaching, tests, and homework designed by the school. Regarding the degree of difficulty of mathematics learning content, it is analyzed from the degree of difficulty of test content, classroom teaching content, homework content, and teaching supplementary materials, as well as the acceptance of the content taught by the school (Valle et al., 2016; Fumero et al., 2020).

By understanding he primary school students’ academic burden from the aspects of the parents, students, and teachers, the reasons for excessive academic burden and primary school students’ mental health problems are analyzed and discussed. The current education system focuses on score competition of examinations, and it only examines students’ memory and the ability to understand relevant knowledge, instead of emphasizing the cultivation of students’ abilities, physical, and mental health. To better count the assessment results, the closed questionnaire was selected. In this questionnaire respondents can only choose from a few answers, such as: always, often, sometimes, and rarely (Manasia et al., 2020).

(1) Basic information on the teacher questionnaire survey. The questionnaire survey was conducted on 98 teachers, including 20 male teachers and 78 female teachers. Seventy-eight teachers had more than 5 years of teaching experience, and 15 teachers had a master’s degree and above. Eighty-seven teachers had primary titles, while 38 teachers had senior titles.
(2) Basic information on the questionnaire survey for primary school students. The questionnaire survey was conducted on 390 primary school students, including 190 male students and 200 female students. For better statistics of the survey results, the students in the third, fourth, and fifth grades with clear self-evaluation ability were selected to conduct the survey, including 120 in the third grade, 130 in the fourth grade, and 140 in the fifth grade.
(3) Basic information on the parent questionnaire survey. The survey was conducted on 260 parents. Questionnaires were randomly distributed to students in the class, including 80 third-grade parents, 80 fourth-grade parents, and 100 fifth-grade parents.

### Reliability and Validity Test of the Questionnaire on Primary School Students’ Academic Stress

Reliability is an index to evaluate the reliability of a questionnaire, and validity is the degree that a measurement tool or method can accurately measure what is needed. The reliability and validity of the data obtained from the questionnaire survey on the same subject was analyzed (Shoahosseini and Baghaei, 2020). The Statistical Product and Service Solutions were used to perform a statistical analysis on the collected questionnaire data. The confirmatory factor analysis was used to test the structural validity. The average variance extracted (AVE) method was adopted to test the discriminant validity, and the Cronbach’s α coefficient was used to test the reliability of the questionnaire. The issuing and collection of questionnaires met the needs of scientific research (Martínez-González et al., 2018).

The KMO value was tested to measure the structural validity of the questionnaire used. The KMO value was 0.967, and the KMO values of the other secondary latent variable dimensions were all above 0.7, indicating that the questionnaire test effect was good and suitable for factor analysis. The AVE method was used to analyze the discriminant validity of the questionnaire. Through calculation, it is known that the AVE value of each dimension is greater than the correlation coefficient among dimensions, that is, the square of the standardized correlation. Therefore, the questionnaires used have discriminant validity among dimensions (Pulido Acosta and Herrera Clavero, 2018).

The Cronbach’s α coefficient was used to process the collected questionnaire data to test the reliability of each dimension of the questionnaire. The calculated Cronbach’s α coefficient was 0.975, and the overall α coefficients of all dimensions were greater than the judgment standard 0.7. It shows high overall internal consistency of the questionnaire data used, good stability, and the overall reliability of the questionnaire, meeting the needs of scientific research.

## Results

The statistics on the questionnaire on the current situation of the mathematics academic burden of primary school students was carried out. The data statistics and analysis of the questionnaire were conducted from the following aspects to accurately introduce the current situation of the mathematics academic burden.

### Understanding of Mathematics Academic Burden

**FIGURE 2:**
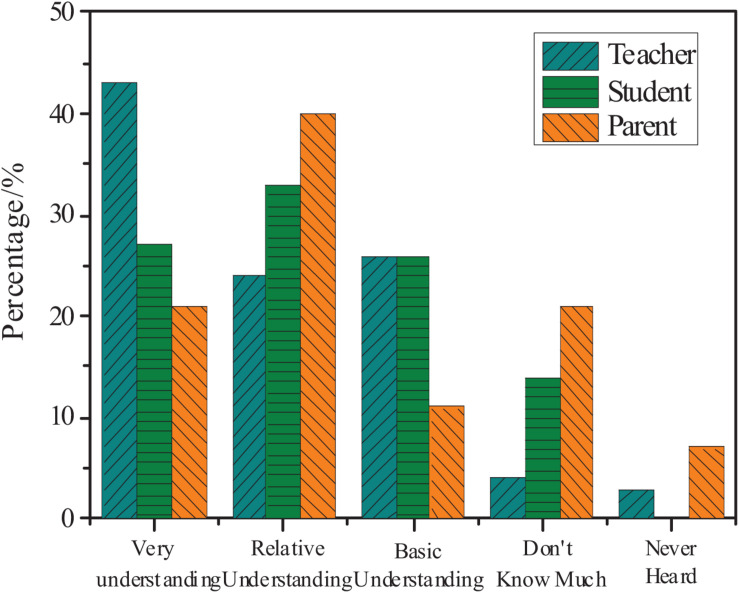
Teachers, parents, and students’ understanding of mathematics academic burden.

The actual understanding of teachers, parents, and students about the mathematics academic burden is counted. The results are shown in Figure 2.

Figure 2 shows that among the students’ understanding of the mathematics academic burden, 61% of them understand very well and relatively well. A total of 40% of parents’ understanding of students’ mathematics academic burden focuses on relative understanding. However, 7% of parents and 3% of teachers have no idea about the mathematics academic burden. In general, teachers have a better understanding of the mathematics academic burden of primary school students.

**FIGURE 3:**
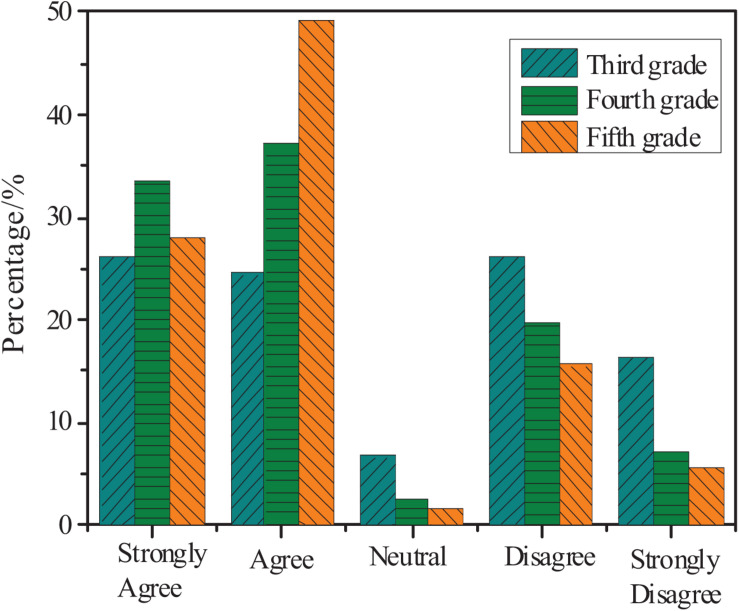
The understanding of mathematics academic burden in different grades.

Figure 3 shows the understanding of the mathematics academic burden in different grades.

Figure 3 shows that in the fifth, fourth, and third grades, the total percentages of *agree* and *strongly agree* are 78, 70, and 50%, respectively. Therefore, even though students are in different grades, most think that they experience a mathematics academic burden.

### Time Investment of Primary School Students in Mathematics

**FIGURE 4:**
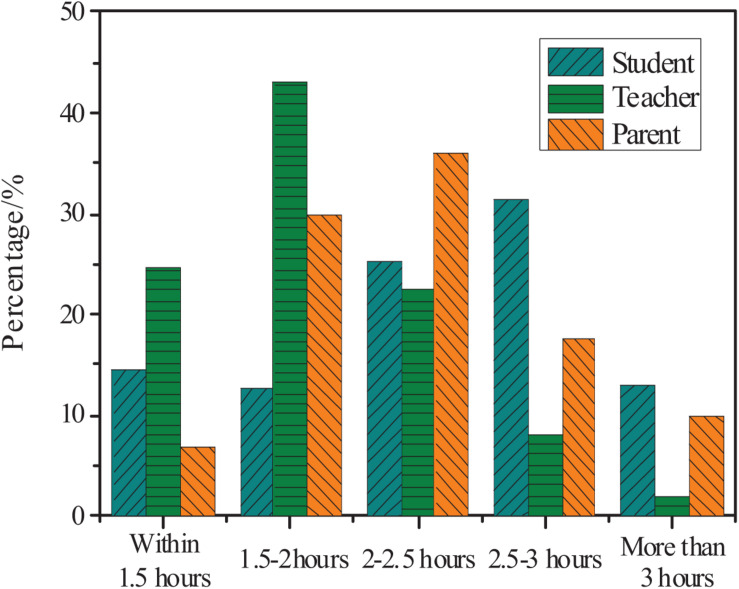
Teachers, parents, and students’ understanding of the daily time spent to complete mathematics homework.

Figure 4 shows the statistics of students, parents, and teachers’ understanding of the current average daily time for primary school students to complete mathematics homework.

Figure 4 shows that 43% of teachers believe that math homework time should be controlled within 1.5–2 h; 35% of parents think the students need 2–2.5 h to complete mathematics homework after school; 30% of students think that it takes 2.5–3 h to complete mathematics homework, and 13% of students need to spend more than 3 h to complete their mathematics homework. According to the regulations of the Ministry of Education, homework for higher grades in primary schools should be limited to 1 h. Therefore, the daily homework of primary school students currently seriously exceeds the time set by the state.

### Average Sleep Time of Primary School Students

**FIGURE 5:**
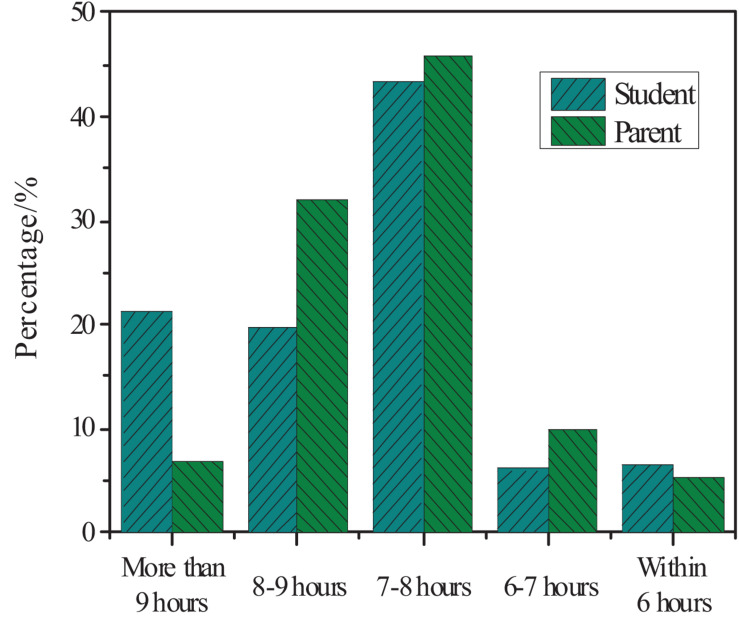
The parents and students’ understanding of the average sleep time of primary school students.

The parents and students’ understanding of the average sleep time of primary school students was counted. The results are shown in Figure 5.

Figure 5 shows that 43% of students think that their sleep time is 7–8 h; 46% of parents think that the sleep time is 7–8 h; 20% of students sleep 8–9 h; 7% of parents believe that the sleep time is more than 9 h. According to the national *Proposals for Physical Fitness*, primary school students should have about 10 h of sleep per day. Compared with the actual sleep time, they generally lack sleep time.

### Extracurricular Activity Time of Primary School Students

**FIGURE 6:**
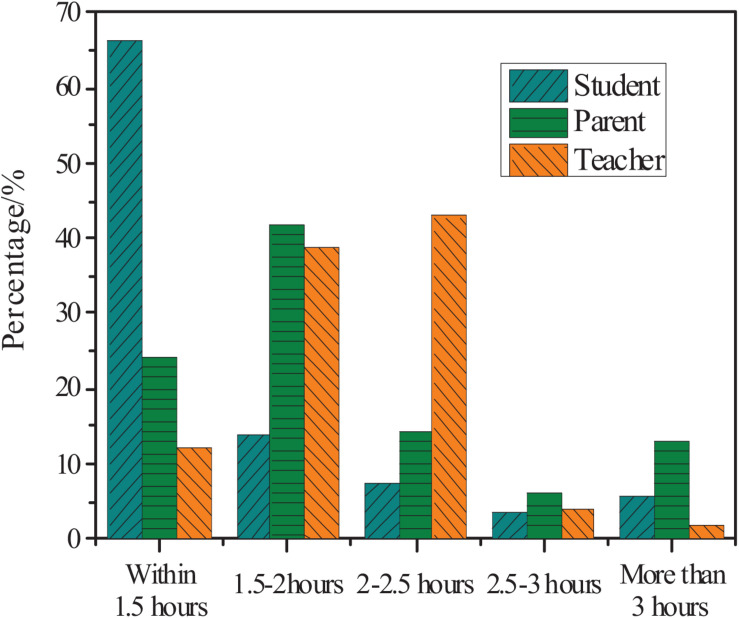
Teachers, parents, and students’ understanding of primary school students’ extracurricular activities time.

From the three aspects of teachers, students, and parents, statistics based on students’ extracurricular activity time were carried out. The results are shown in Figure 6.

Figure 6 shows that 43% of teachers believe that the extracurricular activity time is 2–2.5 h; 42% of parents think that the extracurricular activity time is 1.5–2 h. However, 67% of the students think that the extracurricular activity time is within 1.5 h, and 5.6% of the students think that the extracurricular activity time is more than 3 h. It indicates that the parents do not understand the students’ life and learning, and the teachers may not care too much about extracurricular activities. Thus, there is a big difference in results.

### Learning Content

**FIGURE 7:**
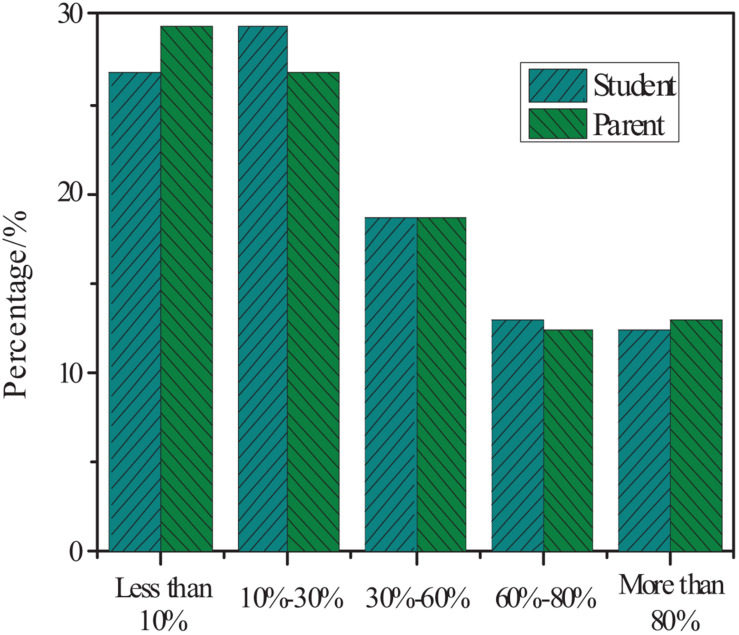
Students and parents’ understanding of the teaching content beyond the knowledge of the textbook.

The statistics on the students and parents’ understanding of the learning content taught by teachers were conducted. The results are shown in Figure 7.

Figure 7 shows that 57% of students and 56% of parents believe that the knowledge that does not belong to the content of the textbook is less than 30%. Therefore, the knowledge content taught by primary school teachers almost meets the school’s curriculum requirements, but there is still some content that falls outside of the curriculum outline. According to the results of the questionnaire, most students have more than two teaching materials. According to national regulations, each person is allowed to have one teaching assistant for subjects that require exams. The results also show that 53% of students take mathematics exams 1–2 times and 29% of students take mathematics exams 3–4 times a month. According to national regulations on the frequency of exams for primary school students, schools can only arrange one final exam for students each semester.

To sum up, in terms of learning time, 42% of students think it takes more than 2.5 h to complete homework, which has a certain impact on their sleep time and extracurricular activity time. For the quality of school work, 57% of students think that part of the course content is not the textbook content. In terms of learning stress, 70% primary school students think that the mathematics burden is heavy. In terms of difficulty, 12% of students need to spend more than 3 h to complete the day’s mathematics homework.

Therefore, the analysis of the survey results of the current students’ mathematical academic burden, show that students, teachers and parents have a general understanding of primary school students’ mathematical academic burden at this stage. A total of 70% of teachers think that primary school students’ mathematical academic burden is heavy; 50% of parents think that primary school students’ academic stress is heavy; 70% of primary school students think that mathematics academic burden is heavy. Such a heavy burden of learning leads to a lack in sleep time and the reduction of extracurricular activity time, which has a serious impact on the physical and mental health of primary school students.

## Discussion

Through the investigation of primary schools in City A, the results show that there is too much mathematics homework, and that part of the learning content taught by teachers falls outside of the curriculum outline, causing students to invest too much time in mathematics learning. Excessive mathematics academic burden causes a decrease in time for sleep and extracurricular activities. The primary school students are both physically and mentally developing. Thinking and perception are selective and comprehensible. If too much time is spent on excessive academics, it will damage their physical and mental health. More students are short-sighted due to long-term bending over to do homework. It has a serious impact on the health of primary school students.

Psychological problems caused by schoolwork burden include cognitive load and emotional load. When primary school students just start to learn the modern knowledge framework, they will gradually become tired of learning about facing many knowledge points with a wide range of content. Also, under the stress of schoolwork, their memory range becomes narrower. The ability to extract the learned knowledge and understand the knowledge is insufficient. The knowledge framework cannot be constructed. Therefore, anxiety will be generated during schoolwork and exams. Primary school students are in the process of personality formation. The ability to deal with complex situations is still immature. Under long-term heavy stress, their expectations will not be met, which will cause anxiety.

Learning anxiety can easily cause mental tension and inner depression in primary school students. If the situation is serious, it will cause students to suffer from mental illnesses such as depression and anxiety, endangering their physical and mental health. It also restricts students’ thinking activities and affects their learning enthusiasm and the teacher-student relationship. Although the country has called for a reduction in the academic burden on students, it is difficult to achieve such a goal in the short term. Therefore, no matter how the environment changes, it is necessary to actively adapt to the changes in the environment. Schools and parents should help students who are still growing to actively face the academic burden. They should be helped to adjust their psychological and emotional attitude toward the academic burden so that they have a positive attitude toward learning. Experienced teachers should improve the training of students’ emotional intelligence in the teaching process and attach importance to their mental health education. Parents should cooperate with the school to establish a comfortable family environment for students, pay more attention to their learning situation at school, and help them solve difficulties in learning and life.

To ensure the physical and mental health development of primary school students, the following solutions are proposed:

1. It is necessary to change teachers’ educational concept so that students are taught with patience, that learning difficulties are correctly understood and that help is provided, and that correct learning attitudes are established. The goal of teaching students should be to provide quality education, to cultivate students’ mathematical literacy, and to improve their ability to solve problems in life.
2. Parents should establish a good family atmosphere for students, encourage students to learn, and establish a confidence in learning well.
3. Students should have a correct learning attitude, face the difficulties encountered in learning, and actively reveal difficulties to parents and teachers.

## Conclusion

The analysis of the PISA report in 2018 shows that the current academic burden of Chinese students is heavy, causing learning anxiety in students. The primary school students’ mathematics homework was selected as the research object. First, the mathematical literacy, mathematics anxiety, and the influencing factors of students’ mathematics anxiety were analyzed. The mathematics academic burden of primary school students was then investigated from three aspects: students, parents, and teachers. The results show that excessive mathematics academic burden reduces sleep time and extracurricular activity time of primary school students, and seriously affects their physical and mental health. However, there are some limitations. The questionnaire designed was not comprehensive enough to conduct a perfect survey of the current situation of primary school students’ mathematics studies. Moreover, the sample areas used were relatively concentrated, which fails to represent the current situation of the mathematics academic burden of all Chinese students. Additionally, the analysis of the questionnaire survey data is also relatively simple. These issues therefore need to be addressed in a follow-up study.

## Data Availability Statement

The raw data supporting the conclusions of this article will be made available by the authors, without undue reservation.

## Ethics Statement

The studies involving human participants were reviewed and approved by People’s Education Press Ethics Committee. Patients/participants provided their written informed consent to participate in this study. Written informed consent was obtained from the individual(s) for the publication of any potentially identifiable images or data included in this article.

## Author Contributions

The author confirms being the sole contributor of this work and has approved it for publication.

## Conflict of Interest

The author declares that the research was conducted in the absence of any commercial or financial relationships that could be construed as a potential conflict of interest.
